# Comparative response of ‘Early Gold’ and ‘Le-Conte’ pear cultivars to dormancy-breaking agents under mild winter conditions

**DOI:** 10.1186/s12870-026-09195-1

**Published:** 2026-06-10

**Authors:** Atef Y. Haleem, Mohamed A. Nasser, Mohamed. K. M. Sayed

**Affiliations:** 1 Plant Production Department, Desert Research Center, Cairo, 11753 Egypt; 2 Department of Horticulture, Faculty of Agriculture, Ain Shams University, 68-Hadayek Shoubra, Cairo, 11241 Egypt; 3 Department of Horticulture, Faculty of Agriculture, New Valley University, El-Kharga, 72511 Egypt

**Keywords:** Hydrogen cyanamide, Mineral oil, Chilling deficiency, Low-chill cultivars, Budburst, Fruit set, Fruit quality

## Abstract

**Background:**

Insufficient winter chilling in warm regions such as Egypt’s New Valley limits budbreak, flowering, and productivity of European pear cultivars. Dormancy‑breaking agents (DBAs), particularly hydrogen cyanamide (HC) and mineral oil (MO), are widely used to overcome these constraints, yet comparative responses among cultivars under arid conditions remain poorly understood. This study evaluated the effects of HC and MO, applied individually or in combination, on the bud behavior, vegetative growth, yield, and fruit quality of two low‑chilling pear cultivars, ‘Early Gold’ and ‘Le‑Conte’, grown under mild winter conditions.

**Results:**

Compared with the untreated control treatment, the dormancy‑breaking treatment significantly improved all the measured traits. Among the tested treatments under the conditions of this study, the combined application of HC 2%+ MO 2% produced the earliest and highest bud break (up to 90.69%), increased the floral bud proportion, and enhanced vegetative growth. HC 2% + MO 2% also resulted in the greatest fruit set (up to 24.14%) and yield (53.62 kg tree⁻¹), followed closely by HC at 3% and the combined HC 1.5% + MO 1.5%. Fruit physical and chemical attributes, including weight, firmness, TSS, and sugar fractions, were markedly improved by these treatments, with HC 2% + MO 2% producing the highest values across seasons. ‘Early Gold’ generally outperformed ‘Le‑Conte’ in terms of fruit set, yield, and quality. Correlation analysis revealed that earlier bud break was strongly associated with greater fruit set, increased yield, and increased sugar accumulation.

**Conclusion:**

Hydrogen cyanamide combined with mineral oil, particularly at 2% each, is highly effective for promoting budbreak, improving yield, and enhancing the fruit quality of pears under inadequate winter chilling. The ‘Early Gold’ cultivar demonstrated superior adaptability to warm conditions, making it a promising cultivar for arid and semiarid production zones. These findings provide practical guidance for optimizing pear management in regions with mild winters.

## Background

European pear (*Pyrus communis* L.) is an economically important fruit crop that is widely cultivated around the world [1, 2]. It is a significant temperate fruit crop, ranking among the top crops globally after apples and grapes. In 2022, global pear production reached 26.32 million tons, with China producing 18 million tons, followed by the United States, Argentina, and Türkiye in fourth position [3]. The area harvested in Egypt was 6,613 ha, with an overall production of about 80,993 tons [3]. Pear is a deciduous fruit tree that requires sufficient winter chilling to establish dormancy, followed by favorable spring temperatures for normal budbreak. Most European pear cultivars (*Pyrus communis* L.) demand high-chilling units, typically 800–1000 chill hours accumulate to ensure timely vegetative budbreak, which is essential for metabolite buildup to support fruit set and development [4–6]. On the other hand, many varieties have low chilling requirements, high yields, and good quality and need to be evaluated under warmer climate conditions [7–9]. The ‘Le-Conte’ pear, a common cultivar in Egypt and many other countries, is a *Pyrus communis* × *Pyrus pyrifolia* hybrid, considered low‑chill cultivars adapted to mild‑winter regions. ‘Le‑Conte’ has a reported chilling requirement of approximately 250–300 chilling hours (≤ 7.2 °C) [10, 11], and is valued for its heat tolerance, pear-shaped fruit, and versatility for fresh consumption or processing [10]. The pear cultivar Early Gold (*Pyrus communis* ‘Early Gold’) originated as an improved seedling of the ‘Ure’ variety and was selected for its enhanced vigor, chlorosis resistance, and superior cold hardiness. whereas the precise chilling requirement of ‘Early Gold’ has not been quantitatively defined; however, it is consistently described as an early‑breaking, low‑chill cultivar [11].

In Egypt, pear cultivation has expanded into newly reclaimed lands characterized by sandy soils and limited water resources, highlighting the need for high-performing and stress-tolerant cultivars [12, 13]. Also, climate change has reduced chilling hours accumulation in production regions [5, 6, 14, 15]. The incidence of bud break normally reaches 50%, and dormancy-breaking agents (DBAs) are therefore applied to mitigate the adverse effects of warm winters. Hydrogen cyanamide (Dormex) is widely used to induce bud dormancy release and enhance flowering and fruit set, often in combination with mineral oil, abscisic acid, gibberellins, or garlic extracts [16, 17]. Dormancy release involves substantial physiological and biochemical changes, including alterations in polyamine metabolism. The levels of polyamines such as putrescine, spermine, spermidine, histamine, and cadaverine reportedly increase during the early stages of dormancy release, reaching peak levels prior to bud break and subsequently declining at full bloom [18].

Hydrogen cyanamide (HC) has been widely studied as an effective dormancy‑breaking agent in deciduous fruit trees, particularly in regions experiencing insufficient winter chilling. Early research in apple (*Malus domestica*) demonstrated that HC accelerates budbreak, enhances flowering uniformity, and improves fruit set by promoting respiratory activity, altering carbohydrate mobilization, and modulating endogenous phytohormone balance during dormancy release [16, 19, 20]. Similar responses have been reported in other pome fruits, including pear (*Pyrus spp.*), although the magnitude of the effect is strongly influenced by species, cultivar, and climatic context [21].

Mineral oil (MO), traditionally used as a dormant spray for pest control [22], has also been shown to influence budbreak by modifying bud microclimate, reducing oxygen diffusion, and promoting ethylene accumulation [23, 24]. When applied alone, MO often results in moderate and less consistent dormancy release compared with HC. However, numerous studies on apple, pear, and stone fruits have demonstrated that combining MO with HC enhances efficacy, allowing reduced HC concentrations while achieving earlier and more uniform budbreak [25–27].

In pear specifically, cultivar‑dependent responses to HC and MO have been documented. Low‑chill cultivars such as ‘Le‑Conte’ and ‘Early Gold’ consistently show improved budbreak, flowering synchronization, and yield when treated with HC alone or in combination with MO under Egyptian and other warm‑climate conditions. Nevertheless, comparative information among low‑chill pear cultivars remains limited, particularly regarding their differential vegetative and reproductive responses, fruit quality outcomes, and sensitivity to combined HC + MO treatments under arid environments. Phenological traits, including bud break, flowering, and fruit set, are critical for pear production, as they determine harvest timing and exposure to adverse weather. Temperature, humidity, and precipitation strongly influence floral synchronization and fruit set under subtropical conditions [28]. The selection of cultivars should consider multiple agronomic traits, including productivity and stability, rather than relying on a single characteristic [29]. Pear floral buds are formed at the termini of shoots and on short spurs aged two years or older; their differentiation is influenced by light exposure, nutrient availability, and pruning practices [30, 31]. Floral differentiation in pear typically occurs during the summer of the preceding growing season, several months before the onset of winter dormancy. During this period, vegetative meristems are induced and transformed into floral meristems, determining the potential number of flowers in the following spring. Successful floral differentiation is therefore a prerequisite for effective dormancy release and subsequent yield improvement, since dormancy-breaking agents can only promote bud break and flowering in buds that have already completed floral initiation [32–34].

The expansion of pear cultivation into Egypt’s newly reclaimed desert lands has created challenges because of insufficient winter chilling. Although hydrogen cyanamide and mineral oil are effective at promoting uniform bud break and enhancing fruit set, their efficiency depends on cultivar, application timing, and environmental conditions [34, 35]. Despite their extensive use, few studies have evaluated the performance of these agents under the arid conditions of the New Valley, and sustainable alternatives remain underexplored.

Accordingly, the present study aims to evaluate the effectiveness of hydrogen cyanamide (Dormex) and mineral oil, applied individually or in combination, on bud dormancy release and fruiting of Early Gold and Le-Conte pear cultivars.

## Materials and methods

### Plant materials

The current research was implemented during two seasons, 2022 and 2023, on pear trees on farms in Afaq in the Balat area, New Valley Governorate, Egypt. with geographical coordinates of 25° and longitude of 29°. The study was conducted in compliance with local laws, and permission was obtained from the farm owner to conduct it. Eight-year-old trees of Early Gold and Le-Conte pear cultivars budded on *P. beutilifolia* rootstock, cultivated in sandy soil at 4 × 6 m apart, and irrigated with drip irrigation were selected. Orchard management practices were standardized, with particular emphasis on fertilization and phytosanitary control through the application of recommended agricultural chemicals.

### Meteorological conditions

**Fig. 1:**
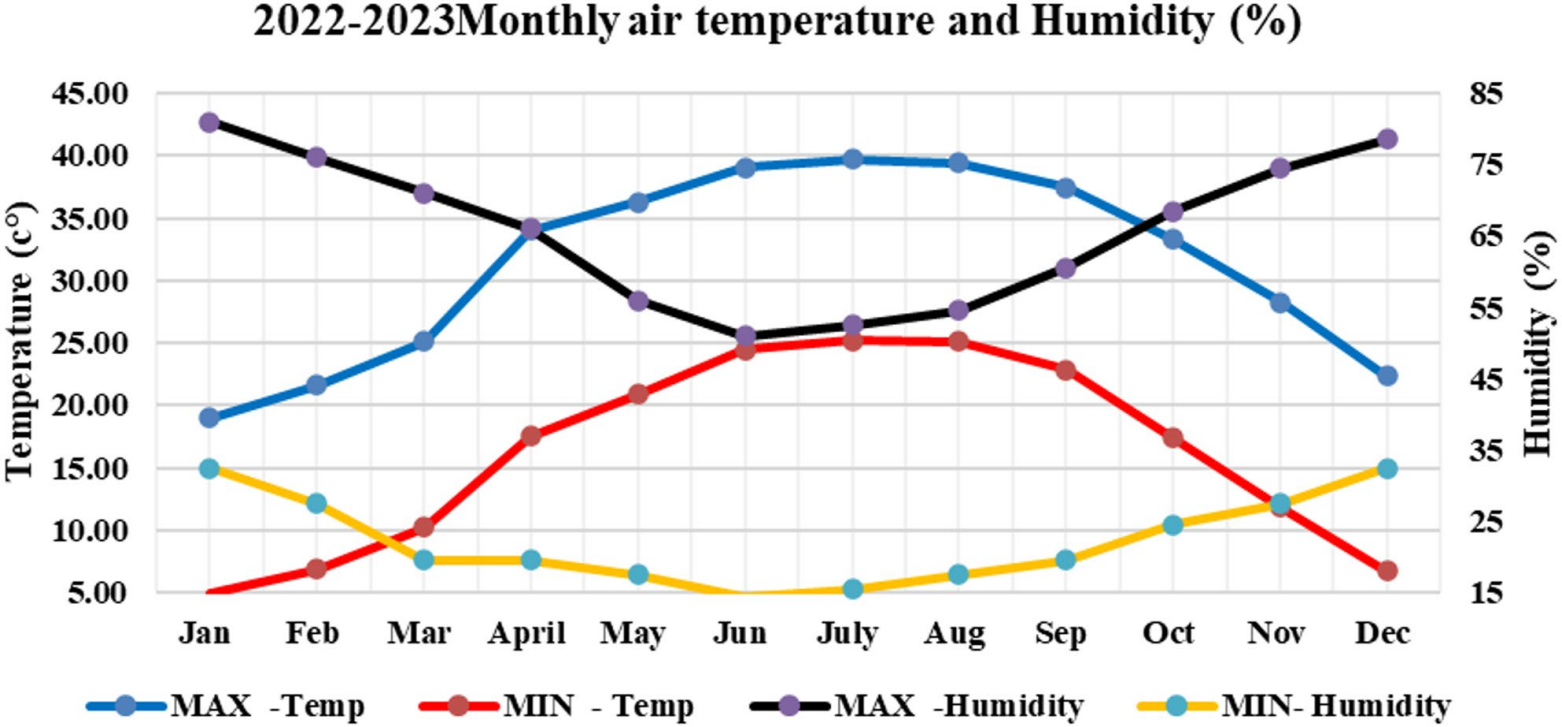
Monthly air temperature and humidity in New Valley, Egypt

The monthly air temperature and relative humidity (%) were recorded during the two studied seasons (2022/2023) using a weather station located in the New Valley region. The data are presented in Figure (1), which illustrates the seasonal variation in climatic conditions. A noticeable decrease in relative humidity and an increase in air temperature was observed from April to September, corresponding to the hot and dry period of the year. Chill accumulation was estimated for the period from November until early February (the spraying date) using two recognized approaches: the Chill Hours model (≤ 7.2 °C) and the Utah model. Based on the Chill Hours model, the accumulated chill reached approximately 225 chilling hours during the 2022 season and 230 chilling hours in 2023. In parallel, chill accumulation was also calculated using the Utah model and expressed as chill units (CU), with values ranging from 150 CU in 2022 and 153 CU in 2023.

### Experimental design

The experiment was designed as a split plot, where the main plots were assigned to pear cultivars, while the subplots were devoted to dormancy-breaking agents, with seven treatments, each of which had three replications, and each replicate was exemplified by one tree.

Forty-two trees were selected for this experiment; all the trees were sprayed with different dormancy breaking agents on February 1st .

The seven spray treatments listed below were used in this experiment:

- Control (water spray only).
- HC 2%: Spraying Hhydrogen cyanamide at 2%.
- HC 3%: Spraying Hhydrogen cyanamide at 3%.
- MO 2%: Spraying Mineral oil at 2%.
- MO 3%: Spraying Mineral oil at 3%.
- HC 1.5% + MO 1.5%: Spraying Hhydrogen cyanamide at 1.5% +Mineral oil at 1.5%.
- HC 2% + MO 2%: Spraying Hhydrogen cyanamide at 2% +Mineral oil at 2%.

Hydrogen cyanamide (Dormex^®^, 49% a.i., Alzchem Group AG, Germany) is a plant growth regulator commonly used in pear trees to break bud dormancy and promote uniform and earlier bud break.

Mineral oil, commonly referred to as horticultural oil or dormant oil, is a highly refined petroleum-derived product composed mainly of saturated hydrocarbons. It typically contains 70–90% paraffinic hydrocarbons (C15–C30 alkanes), 5–25% naphthenic hydrocarbons (cycloalkanes), and less than 1% aromatic hydrocarbons, along with small amounts of emulsifiers and stabilizers, to ensure miscibility with water. Dormancy-breaking treatments, including hydrogen cyanamide (Dormex), mineral oil, and their combinations, were applied using a calibrated motorized orchard sprayer equipped with hollow-cone nozzles. A spray volume of approximately 5 L of tree was used, equivalent to 2085 L ha⁻¹ based on a planting density of 417 trees ha⁻¹ (4 × 6 m spacing). All applications were performed to runoff to ensure thorough coverage of dormant buds and scaffold branches. Spraying was conducted during the early morning hours under calm, low-wind conditions, with ambient temperatures ranging between 7 and 22 °C, to minimize evaporation and spray drift. Furthermore, no precipitation occurred within 48 h post application, ensuring optimal chemical absorption and preventing wash-off.

### Measurements

Behavior of buds: The number of days from the application of dormancy-breaking agents to bud break was recorded. In each growing season, one main branch was selected from each of the four cardinal directions (north, south, east, and west) of every experimental tree. The selected branches were as uniform as possible and tagged, and the bud break percentage was recorded. Bud break was defined as the phenological stage at which bud scales separate and the first green tissue becomes visible (BBCH stage 07) [36]. In addition, the percentage of vegetative and floral buds relative to the total number of bud breaks per branch [37].

 Vegetative growth traits, i.e., main shoot length (cm), number of leaves/shoot and leaf area (cm^2^), were measured. The leaf area per leaf (cm^2^) was measured according to [38] Eq. (1).

1 $$\:Leaf\:area=0.69\:\times\:\:\left(LW\right)$$

L: length of longest leaf; W: width of widest leaf.

The average leaf area per tree was subsequently calculated based on a representative sample of leaves.

### Fruit set and yield

The percentage of fruit set was calculated by dividing the number of fruits set at the pea stage per shoot by the number of hermaphrodite flowers per shoot and is expressed in percentage Eq. (2).

2 $$\:Fruit\:set\:\%=\:\frac{Number\:of\:fruit\:set}{Total\:EquationNumber\:of\:perfect\:flowers}*100$$

At optimal maturity in August [39–41], a random selection of ten fruits from each treatment was sampled. The yield per tree (kg) was calculated by multiplying the average fruit weight (g) by the total number of fruits per tree after the fruits were harvested, and their weight (g) was noted.

### Fruit physical characteristics

Fruit weight (g), fruit length (cm), fruit diameter (cm), fruit shape index (length/diameter, L/D), and fruit firmness (lb/inch^2^) were measured using a penetrometer (pressure tester) Advance Force Gauge RH 13, UK.

### Fruit chemical characteristics

Juice was extracted from ten fruits, and the TSS content was determined as °Brix using a digital refractometer (Model PR-32, Atago, Japan). Total acidity (TA) was assessed as malic acid by titration with 0.1 N sodium hydroxide solution using phenolphthalein as an indicator, following AOAC procedures [42]. The percentage of acidity was calculated using Eq. (3).

3 $$\:Total\:acidity\%=\:\frac{0.0075*NaoH\:\left(mL\right)* Normality of NaoH}{juice\:volume\:\left(mL\right)}\mathrm{*}100\:$$

The TSS/acid ratio was calculated, and the sugar content% and vitamin C content were determined in (10 g) fruit pulp extracted with 3% oxalic acid to inhibit oxidation. The extract was then titrated against a 2,6-dichlorophenolindophenol solution until a persistent pink endpoint. The iodometric titration method was used to calculate the percentages of total sugars, reduced sugars, and nonreduced sugars [43].

### Statistical analysis

The experiment was arranged in a split-plot design with three replications. The data were subjected to split‑plot analysis of variance (ANOVA) according to the procedures described by Steel and Torrie [44]. Cultivars were tested against the main‑plot error, whereas dormancy‑breaking agents (DBAs) and the cultivar × DBA interaction were tested against the subplot error. Statistical analyses were performed using SAS software (version 8.2). Analysis of variance (ANOVA) was conducted using the PROC ANOVA procedure, and mean comparisons were performed with Tukey’s multiple comparison test at a significance level of α = 0.05, which was based on the appropriate error term for each factor in the split‑plot design. The results are presented as the mean ± standard error (SE), and statistical significance was considered at *p* < 0.05. Heatmap correlation analysis was conducted using R Studio software with the “metan” package to examine the relationships among bud break time (days from spraying to bud break), fruit set percentage, yield, and fruit quality traits. The percentage change was calculated according to the following equation$\%\:\mathrm{Change}\:\text {vs.}\:\text {Control }=\frac{\text{(Treatment-Control value)}}{\mathrm{Control}\:\mathrm{value}}{\times\:100}$ .

## Results

### Bud behavior

**Fig. 2:**
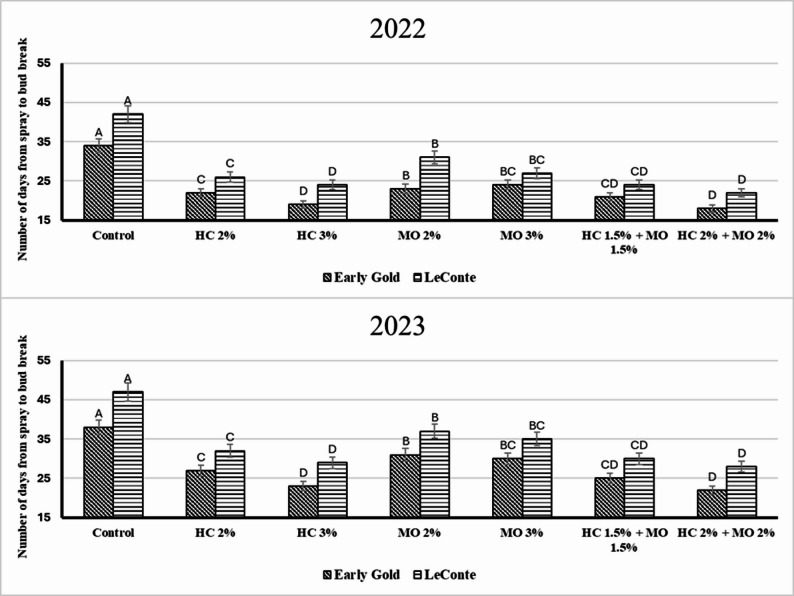
Effect of spraying hydrogen cyanamide and mineral oil on the number of days from spray dormancy-breaking spray applications (1 February) to bud break of Early Gold and Le-Conte pear cultivars during 2022 and 2023 seasons

**Fig. 3:**
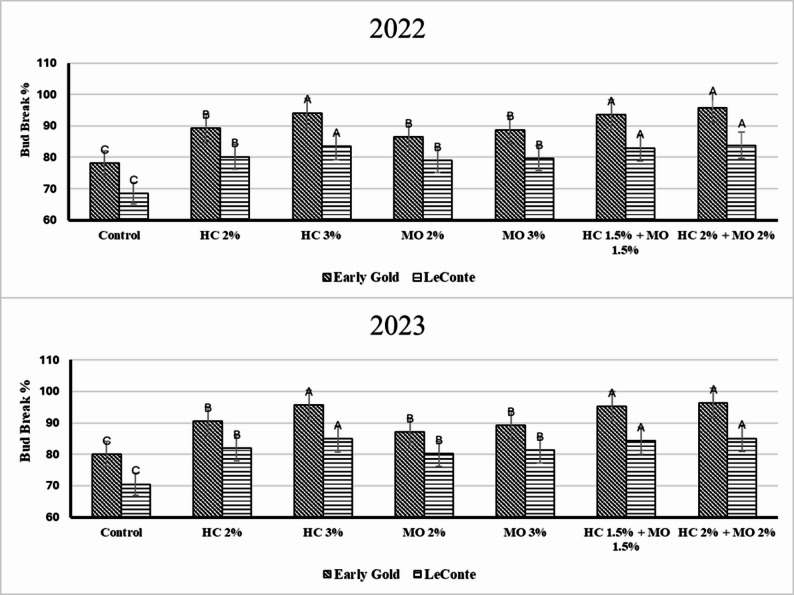
Effect of spraying hydrogen cyanamide and mineral oil on bud break of Early Gold and Le-Conte pear cultivars during 2022 and 2023 seasons

**Fig. 4:**
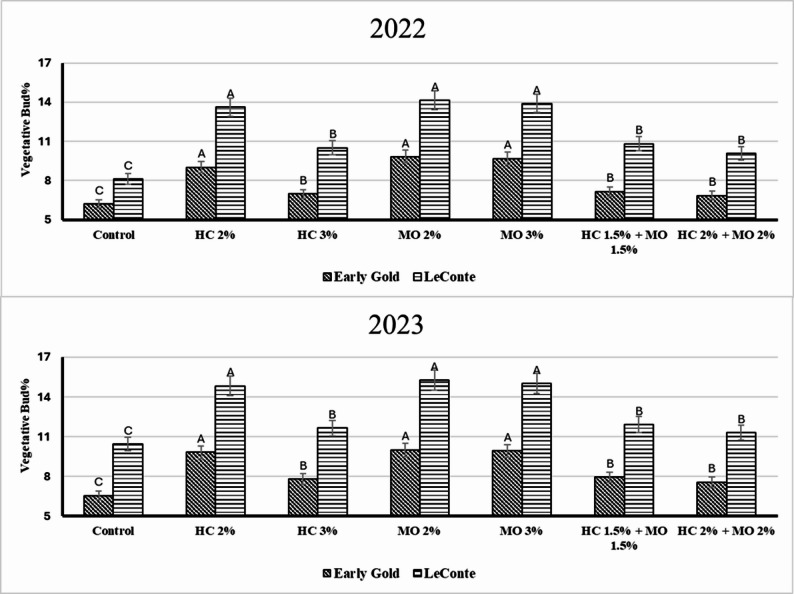
Effect of spraying hydrogen cyanamide and mineral oil on the percentage of vegetative buds of the Early Gold and Le-Conte pear cultivars during 2022 and 2023 seasons

**Fig. 5:**
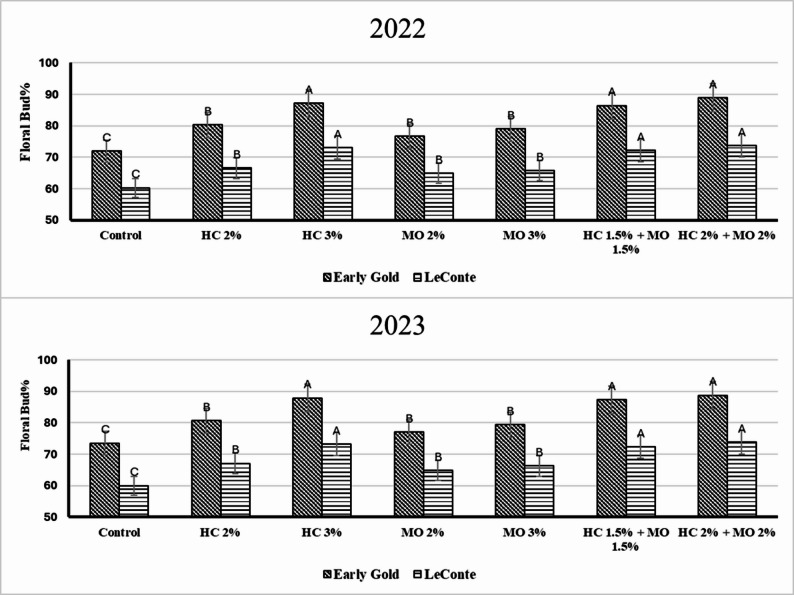
Effect of spraying hydrogen cyanamide and mineral oil on the percentage of floral buds of the Early Gold and Le-Conte pear cultivars during 2022 and 2023 seasons

Bud behavior varies depending on cultivar and dormancy-breaking treatment. Figures (2, 3, 4 and 5) present the impact of spraying hydrogen cyanamide and mineral oil on bud behavior, including the number of days from spray to bud breaks, the bud break percentage, and the proportion of vegetative and floral buds in Early Gold and Le-Conte pear cultivars during the 2022 and 2023 seasons. Significant differences were observed in the percentage of bud break, and the percentages of vegetative and floral buds were influenced by the applied dormancy-breaking agents and the two cultivars throughout the study period.

Among the evaluated treatments, HC 2% + MO 2% resulted in the highest values of bud break number, bud break percentage, and floral bud proportion, followed by HC 3% and HC 1.5% + MO 1.5%. The plants sprayed with water (control) presented the minimum value. The corresponding increases in bud break over the control were 18.33% and 16.98%, whereas the increases in floral bud percentage reached 18.69% and 17.81% during the two seasons, respectively.

The recorded values of Bud break were 73.34, 84.75, 88.83, 82.81, 84.25, 88.30 and 89.80 & 75.29, 86.19, 90.28, 83.65, 85.29, 89.79 and 90.69%, and those of Floral bud were 66.13, 73.43, 80.08, 70.82, 72.54, 79.30 and 81.33 & 66.79, 73.87, 80.55, 71.02, 72.82, 79.87 and 81.26%, respectively. of the two seasons studied, respectively) due to control, HC 2%, HC 3%, MO 2%, MO 3%, HC 1.5% + MO 1.5% and HC 2% + MO 2%, respectively. The corresponding increment of bud break over the control was 18.33 and 16.98%, and the percentage of Floral bud was 18.69 and 17.81% during the two seasons studied.

### Vegetative growth traits

**Fig. 6:**
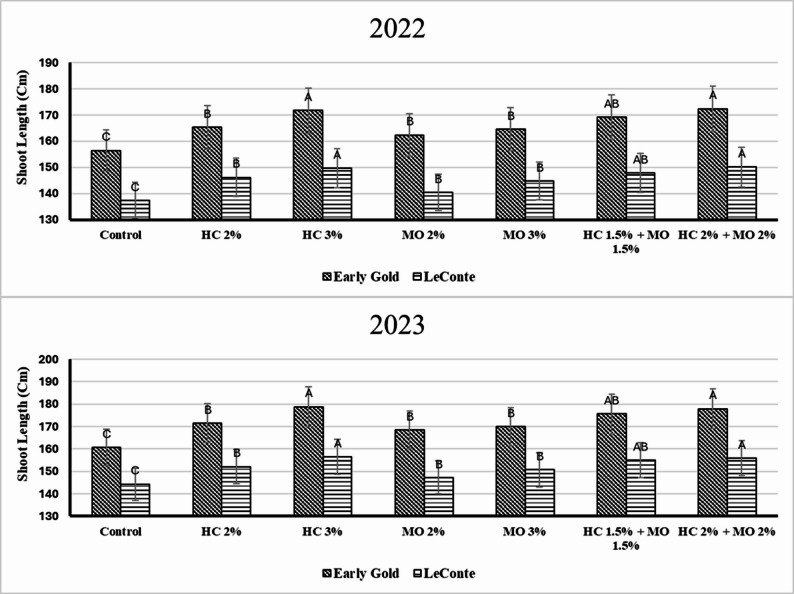
Effect of spraying hydrogen cyanamide and mineral oil on the shoot length (cm) of Early Gold and Le-Conte pear cultivars during 2022 and 2023 seasons

**Fig. 7:**
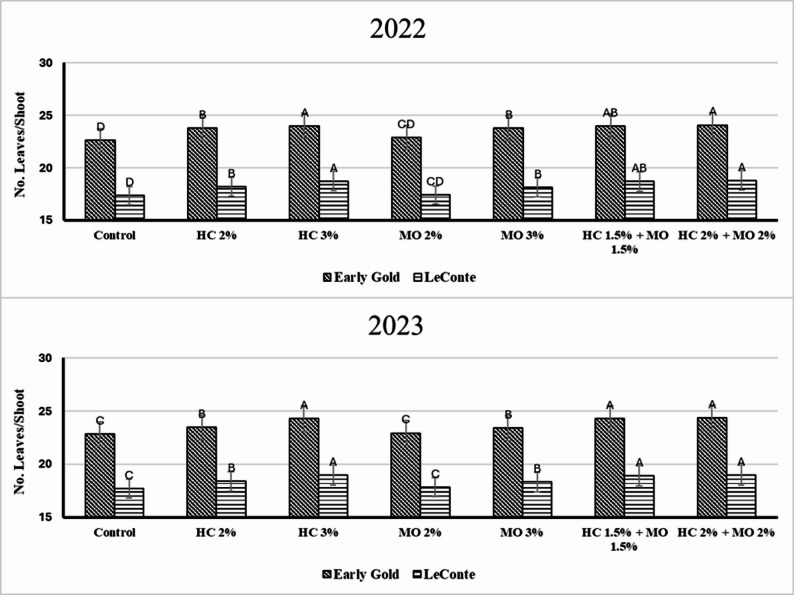
Effect of spraying hydrogen cyanamide and mineral oil on the number of leaves/shoots of the Early Gold and Le-Conte pear cultivars during 2022 and 2023 seasons

**Fig. 8:**
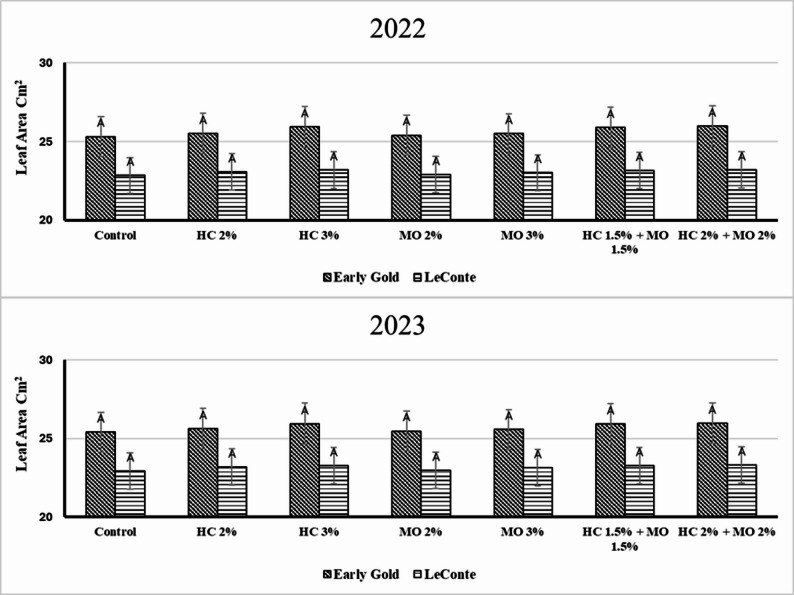
Effect of spraying hydrogen cyanamide and mineral oil on the leaf area cm^2^ of the Early Gold and Le-Conte pear cultivars during 2022 and 2023 seasons

With respect to dormancy-breaking agents, the data presented in Figures (6, 7 and 8) indicate that treatment HC 2% + MO 2% presented the greatest shoot growth parameters across both seasons. Specifically, HC 2% + MO 2% resulted in shoot lengths of 173 cm and 179 cm, accompanied by 21.44 and 21.69 leaves per shoot, and leaf areas of 24.59 cm² and 24.65 cm² during the first and second seasons, respectively. These values closely followed those of HC 3% (172 cm and 180 cm shoot length; 21.37 and 21.65 leaves per shoot; 24.56 cm² and 24.61 cm² leaf area) and HC 1.5% + MO 1.5% (170 cm and 173 cm shoot length; 21.33 and 21.61 leaves per shoot; 24.52 cm² and 24.59 cm² leaf area). In contrast, the control treatment, where the plants were sprayed with water, *presented* the lowest values, with shoot lengths of 155 cm and 160 cm, 19.99 and 20.27 leaves per shoot, and leaf areas of 24.08 cm² and 24.16 cm² in the first and second seasons, respectively. Therefore, the corresponding increase in shoot length over the control was 8.91 and 8.61%, the number of leaves per shoot was 6.76 and 6.55%, and the leaf area was 2.07 and 1.99% during the two seasons studied.

The results revealed that the shoot length, number of leaves per shoot, and leaf area were significantly influenced by cultivar across all the treatments. The ‘Early Gold’ cultivar presented the highest values for both the number of leaves per shoot and the leaf area, with 23.59 and 23.66 leaves per shoot and 25.65 cm² and 25.70 cm² leaf areas during the first and second seasons, respectively. In contrast, the ‘Le-Conte’ cultivar produced the lowest values, with 18.19 and 18.45 leaves per shoot and 23.05 cm² and 23.15 cm² leaf areas in the different seasons. Consequently, the percentage increase in the number of leaves per shoot for ‘Early Gold’ compared with ‘Le-Conte’ was 22.89% and 22.02%, whereas the increase in the leaf area was 10.14% and 9.92% during the two seasons, respectively.

### Fruit set and yield

The fruit set and yield of the two pear cultivars were significantly influenced by the application of different dormancy-breaking agents during both seasons (Table 1). The highest fruit set percentages were recorded with the HC 2% + MO 2% treatment (22.79% and 21.23%), followed by the HC 3% treatment (22.33% and 23.95%) and HC 1.5% + MO 1.5% treatment (22.11% and 23.68%) in the first and second seasons, respectively. Conversely, the control treatment presented the lowest fruit set values (8.81% and 9.25%). A similar trend was observed for yield per tree, where HC 2% + MO 2% achieved the maximum yield (51.41 kg and 53.62 kg), followed by HC 3% (50.90 kg and 52.85 kg) and HC 1.5% + MO 1.5% (50.14 kg and 52.08 kg), whereas the control presented the minimum yield (40.19 kg and 41.11 kg) across the two seasons. Therefore, the corresponding increase in yield per tree over the control was 21.82% and 23.33% during the two seasons studied.

**Table 1:** Effect of spraying hydrogen cyanamide and mineral oil on fruit set and yield per tree of Early Gold and Le-Conte pear cultivars during 2022 and 2023 seasons

Treatments	Cultivars					
	Fruit Set %			Yield per tree (kg)		
	Early Gold	Le-Conte	Mean	Early Gold	Le-Conte	mean
2022						
control	10.92 m	6.70n	8.81G	43.73j	36.64n	40.19G
HC 2%	21.85d	14.43j	18.14D	48.89d	40.36k	44.63D
HC 3%	25.48b	19.17 h	22.33B	56.93b	44.87 h	50.90B
MO 2%	21.26f	11.56 L	16.41 F	47.81f	39.16 m	43.49 F
MO 3%	21.37e	12.20k	16.79E	48.38e	39.91 L	44.15E
HC 1.5% + MO 1.5%	25.24c	18.97i	22.11 C	56.2c	44.08i	50.14 C
HC 2% + MO 2%	25.81a	19.76 g	22.79 A	57.29a	45.52 g	51.41 A
mean	21.71 A	14.68B		51.32 A	41.51B	
2023						
control	11.66 m	6.83n	9.25G	44.61j	37.61n	41.11 F
HC 2%	22.91d	15.68j	19.30D	49.77f	41.73k	45.75D
HC 3%	26.89b	21.00 h	23.95B	58.82b	46.88 h	52.85B
MO 2%	22.17f	12.71 L	17.44 F	50.03d	41.02 m	45.53E
MO 3%	22.65e	13.08k	17.87E	49.91e	41.55 L	45.73D
HC 1.5% + MO 1.5%	26.78c	20.57i	23.68 C	57.97c	46.18i	52.08 C
HC 2% + MO 2%	27.04a	21.23 g	24.14 A	59.61a	47.63 g	53.62 A
mean	22.87 A	15.87B		52.96 A	43.23B	

Means in the same column followed by the same letter (s) are not significantly (*p* ≥ 0.05) different. according to Tukey’s multiple range test. Comparisons among cultivars were performed using the main‑plot error term, whereas comparisons among dormancy‑breaking agent treatments and their interactions with cultivars were performed using the sub‑plot error term

Control (water spray only), HC 2%: Spraying Hydrogen cyanamide at 2%, HC 3%: Spraying Hydrogen cyanamide at 3%, MO 2%: Spraying Mineral oil at 2%, MO 3%: Spraying Mineral oil at 3%, HC 1.5% MO 1.5%: Spraying Hydrogen cyanamide at 1.5% +Mineral oil at 1.5%, HC 2% + MO 2%: Spraying Hydrogen cyanamide at 2% +Mineral oil at 2%

The findings showed that cultivars had a significant effect on fruit set and yield per tree in all the treatments. In the first and second seasons, respectively, the “Early Gold” cultivar consistently yielded the highest fruit set (21.70% and 22.87%) and yield per tree (51.32 kg and 52.96 kg). Le-Conte, on the other hand, had the lowest yield per tree values (41.51 kg and 43.23 kg) and fruit set (14.68% and 15.87%). As a result, “Early Gold” had a yield advantage over “Le-Conte” of 19.12% and 18.37%, respectively, during the two seasons.

### Fruit physical characteristics

Tables 2 and 3 present the effects of spraying hydrogen cyanamide and mineral oil on the fruit characteristics of Early Gold and Le-Conte pear cultivars during the 2022 and 2023 seasons. The data clearly revealed that the fruit characteristics followed a similar trend across the two experimental seasons. Fruit weight, fruit dimensions and fruit firmness significantly differed between the different treatments and the two cultivars.

**Table 2:** Effect of spraying hydrogen cyanamide and mineral oil on the fruit diameter, fruit length and fruit shape of Early Gold and Le-Conte pear cultivars during 2022 and 2023 seasons

Treatments	Cultivars								
	Fruit diameter (cm)			Fruit length (cm)			Fruit shape		
	Early Gold	Le-Conte	mean	Early Gold	Le-Conte	mean	Early Gold	Le-Conte	mean
2022									
control	5.64c	5.02 g	5.33B	6.92de	6.21 g	6.57D	1.23f	1.24e	1.24 C
HC 2%	5.89bc	5.22d-g	5.56B	7.52a-c	6.47e-g	7.00BC	1.28a	1.24e	1.26B
HC 3%	6.32a	5.54c-e	5.93 A	7.96a	6.73ef	7.34AB	1.26c	1.22 h	1.24 C
MO 2%	5.77c	5.10 fg	5.44B	7.37 cd	6.36 fg	6.87CD	1.28a	1.25d	1.27 A
MO 3%	5.83c	5.17e-g	5.50B	7.42bc	6.40 fg	6.91CD	1.27b	1.24e	1.26B
HC 1.5% + MO 1.5%	6.28ab	5.49c-f	5.89 A	7.89ab	6.68e-g	7.29AB	1.26c	1.22 g	1.24 C
HC 2% + MO 2%	6.40a	5.61 cd	6.01 A	8.00a	6.80ef	7.40 A	1.25d	1.21i	1.23D
mean	6.02 A	5.31B		7.58 A	6.52B		1.26 A	1.23B	
2023									
control	5.86d-f	5.13i	5.50E	7.37de	6.37f	6.87 C	1.26 g	1.24 h	1.25E
HC 2%	6.28a-c	5.44 g-i	5.86B-D	8.03a-c	6.97e	7.50B	1.28d	1.28d	1.28B
HC 3%	6.54ab	5.68e-h	6.11AB	8.33ab	7.83b-d	8.08 A	1.27f	1.38b	1.33 A
MO 2%	6.07c-e	5.31hi	5.69DE	7.84b-d	6.86ef	7.35B	1.29c	1.29c	1.29B
MO 3%	6.19b-d	5.37 g-i	5.78 C-E	7.93a-c	6.90ef	7.42B	1.28d	1.29c	1.29B
HC 1.5% + MO 1.5%	6.47a-c	5.62f-h	6.04 A-C	8.26a-c	7.78 cd	8.02 A	1.28d	1.39a	1.34 A
HC 2% + MO 2%	6.62a	5.74e-g	6.18 A	8.44a	7.93a-c	8.19 A	1.28d	1.38b	1.33 A
mean	6.29 A	5.47B		8.03 A	7.24B		1.28B	1.32 A	

Means in the same column followed by the same letter (s) are not significantly (*p* ≥ 0.05) different. according to Tukey’s multiple range test. Comparisons among cultivars were performed using the main‑plot error term, whereas comparisons among dormancy‑breaking agent treatments and their interactions with cultivars were performed using the sub‑plot error term

Control (water spray only), HC 2%: Spraying Hydrogen cyanamide at 2%, HC 3%: Spraying Hydrogen cyanamide at 3%, MO 2%: Spraying Mineral oil at 2%, MO 3%: Spraying Mineral oil at 3%, HC 1.5% MO 1.5%: Spraying Hydrogen cyanamide at 1.5% +Mineral oil at 1.5%, HC 2% + MO 2%: Spraying Hydrogen cyanamide at 2% +Mineral oil at 2%

**Table 3:** Effect of spraying hydrogen cyanamide and mineral oil on the fruit weight and fruit firmness of Early Gold and Le-Conte pear cultivars during 2022 and 2023 seasons

Treatments	Cultivars					
	Fruit weight (g)			Fruit firmness lb/inch^2^		
	Early Gold	Le-Conte	mean	Early Gold	Le-Conte	mean
2022						
control	115.56bc	101.51d	108.54 C	14.75b	10.87c	12.82B
HC 2%	125.00a	109.50 cd	117.25AB	15.96a	13.93b	14.94 A
HC 3%	128.40a	113.37c	120.89AB	16.21a	14.25b	15.23 A
MO 2%	123.94ab	107.89 cd	115.92B	15.84a	13.78b	14.81 A
MO 3%	124.73a	108.74 cd	116.74AB	15.90a	13.86b	14.88 A
HC 1.5% + MO 1.5%	127.87a	111.92c	119.90AB	16.18a	14.19b	15.18 A
HC 2% + MO 2%	130.21a	115.61bc	122.91 A	16.29a	14.31b	15.30 A
mean	125.10 A	109.79B		15.87 A	13.60B	
2023						
control	120.33c	106.92d	113.63D	15.68b-d	12.77e	14.23B
control	149.56b	117.94c	133.75 A-C	16.22ab	15.16b-d	15.69 A
HC 2%	157.08ab	120.21c	138.65AB	16.92a	15.38b-d	16.15 A
HC 3%	148.69b	113.86 cd	131.28 C	16.07a-c	14.92d	15.50 A
MO 2%	148.95b	114.69 cd	131.82BC	16.16ab	15.03 cd	15.60 A
MO 3%	155.89ab	119.74c	138.82 A-C	16.85a	15.30b-d	16.08 A
HC 1.5% + MO 1.5%	160.05a	121.11c	140.58 A	17.07a	15.47b-d	16.27 A
mean	148.65 A	116.35B		16.42 A	14.86B	

Means in the same column followed by the same letter (s) are not significantly (*p* ≥ 0.05) different. according to Tukey’s multiple range test. Comparisons among cultivars were performed using the main‑plot error term, whereas comparisons among dormancy‑breaking agent treatments and their interaction with cultivars were performed using the sub‑plot error term

Control (water spray only), HC 2%: Spraying Hydrogen cyanamide at 2%, HC 3%: Spraying Hydrogen cyanamide at 3%, MO 2%: Spraying Mineral oil at 2%, MO 3%: Spraying Mineral oil at 3%, HC 1.5% MO 1.5%: Spraying Hydrogen cyanamide at 1.5% +Mineral oil at 1.5%, HC 2% + MO 2%: Spraying Hydrogen cyanamide at 2% +Mineral oil at 2%

The maximum values of fruit weight, fruit length, fruit diameter and fruit firmness occurred at HC 2% + MO 2%, followed by those HC 3% and HC 1.5% + MO 1.5%. The spraying water (control) trees presented the minimum values of fruit weight, fruit length, fruit diameter and fruit firmness during the two studied seasons.

The highest values of fruit weight (123.13, 121.10 and 120.12 g) were obtained by HC 2% + MO 2%, HC 3% and HC 1.5% + MO 1.5%, respectively, in the first season and 140.83, 138.90 and 138.07 g, respectively, in the second season. Therefore, the corresponding increase in fruit weight over the control was 11.69 and 19.17%, respectively. In terms of fruit length, HC 2% + MO 2% presented the greatest significant values (7.40 and 8.19 cm) in the two seasons, followed by HC 3% (7.34 and 8.08 cm) and HC 1.5% + MO 1.5% (7.29 and 8.02 cm) in the two seasons. In terms of fruit diameter, the greatest values were obtained by HC 2% + MO 2% (6.01 and 6.18 cm) in the two seasons, followed by HC 3% (5.93 and 6.11 cm) and HC 1.5% + MO 1.5% (5.89 and 6.05 cm) in the two seasons. The firmness of the fruit increased, especially in the second season, by HC 2% + MO 2% (16.27 lb/inch^2^), followed by HC 3% (16.15 lb/inch^2^) and HC 1.5% + MO 1.5% (8.02 lb/inch^2^).

The Early Gold cultivar outperformed the Le-Conte cultivar in terms of fruit physical characteristics, with the highest values for fruit weight, fruit length, fruit diameter and fruit firmness. The greatest values of fruit weight and fruit firmness were observed for the Early Gold cultivar, whose values were 125.33 and 148.91 cm and 15.88 and 16.43 lb/inch^2^ during the two studied seasons, respectively. The Le-Conte cultivar presented the minimum values, which were 109.99 and 116.56 cm and 13.60 and 14.46 lb/inch^2^ during the two studied seasons, respectively. Hence, the increase in fruit weight over the control was 12.24% and 21.72%, and fruit firmness was 14.36% and 9.56% during the two seasons studied.

### Fruit chemical characteristics

The highest values of TSS and the TSS/acid ratio were obtained when trees were subjected to HC 2% + MO 2% (12.29 and 46.33) in the first season and (12.71 and 47.92) in the second season, followed closely by those subjected to HC 1.5% + MO 1.5% and HC 3% (Table 4). On the other hand, in comparison with Le-Conte, Early Gold presented the best values of TSS (12.23 and 12.50 °Brix) and the highest TSS/acid ratio (45.30 and 46.30) in the two seasons. There was no significant effect of treatment or cultivar on acidity%.

**Table 4:** Effect of spraying hydrogen cyanamide and mineral oil on the percentage of total soluble solids (TSS), acidity, and the TSS/acid ratio of Early Gold and Le-Conte pear cultivars during 2022 and 2023 seasons

Treatments	Cultivars								
	TSS °Brix			Acidity %			TSS/acid ratio		
	Early Gold	Le-Conte	mean	Early Gold	Le-Conte	mean	Early Gold	Le-Conte	mean
2022									
control	11.09 fg	10.32 g	10.70B	0.280a-c	0.287a	0.283 A	39.76j	35.83k	37.79G
HC 2%	12.28a-c	11.40d-f	11.84 A	0.270a-c	0.283ab	0.278 A	45.49d	40.57i	43.03D
HC 3%	12.62a	11.78b-f	12.20 A	0.257c	0.277a-c	0.267 A	49.13b	42.39gh	45.76B
MO 2%	12.16a-e	11.27f	11.72 A	0.277a-c	0.287a	0.282 A	43.92f	39.54j	41.73 F
MO 3%	12.21a-d	11.33ef	11.77 A	0.273a-c	0.283ab	0.278 A	44.75e	39.90j	42.33E
HC 1.5% + MO 1.5%	12.56ab	11.71c-f	12.13 A	0.260bc	0.277a-c	0.268 A	48.14c	42.12 h	45.13 C
HC 2% + MO 2%	12.70a	11.87a-f	12.29 A	0.257c	0.277a-c	0.267 A	49.83a	42.88 g	46.36 A
Mean	12.23 A	11.38B		0.268B	0.281 A		45.86 A	40.46B	
2023									
control	11.66d	10.70e	11.18 C	0.283a-c	0.297a	0.290 A	41.07 h	36.15j	38.61G
HC 2%	12.38a-d	12.05 cd	12.22AB	0.277a-c	0.290ab	0.283 A-C	44.87d	41.84 g	43.36D
HC 3%	12.97ab	12.29a-d	12.63AB	0.267c	0.280a-c	0.273BC	48.96b	44.06e	46.51B
MO 2%	12.22b-d	11.88d	12.05B	0.280a-c	0.293a	0.287AB	43.51f	40.55i	42.03 F
MO 3%	12.30a-d	11.92d	12.11AB	0.280a-c	0.293a	0.287AB	44.26e	40.84 h	42.55E
HC 1.5% + MO 1.5%	12.89a-c	12.21b-d	12.55AB	0.270bc	0.280a-c	0.275 A-C	48.11c	43.47f	45.79 C
HC 2% + MO 2%	13.10a	12.32a-d	12.71 A	0.263c	0.277a-c	0.270 C	49.83a	44.64d	47.23 A
Mean	12.51 A	11.91B		0.274B	0.287 A		45.80 A	41.65B	

Means in the same column followed by the same letter (s) are not significantly (*p* ≥ 0.05) different. according to Tukey’s multiple range test. Comparisons among cultivars were performed using the main‑plot error term, whereas comparisons among dormancy‑breaking agent treatments and their interactions with cultivars were performed using the sub‑plot error term

Control (water spray only), HC 2%: Spraying Hydrogen cyanamide at 2%, HC 3%: Spraying Hydrogen cyanamide at 3%, MO 2%: Spraying Mineral oil at 2%, MO 3%: Spraying Mineral oil at 3%, HC 1.5% MO 1.5%: Spraying Hydrogen cyanamide at 1.5% +Mineral oil at 1.5%, HC 2% + MO 2%: Spraying Hydrogen cyanamide at 2% +Mineral oil at 2%

The total sugars %, reducing sugars %, and non-reducing sugars % were affected by the spraying treatments and cultivar (Table 5). HC 2% + MO 2% had the highest values of total sugars %, reducing sugars %, and non-reducing sugars % (10.12, 5.90, and 4.37, respectively) in the first season and (10.49, 6.12, and 4.37, respectively) in the two seasons respectively.

**Table 5:** Effect of spraying hydrogen cyanamide and mineral oil on percentage of total, reducing and non-reducing sugars in the Early Gold and Le-Conte pear cultivars during 2022 and 2023 seasons

Treatments	Cultivars								
	Total sugars %			Reducing sugars %			Non-reducing sugars (%)		
	Early Gold	Le-Conte	Mean	Early Gold	Le-Conte	mean	Early Gold	Le-Conte	mean
2022									
control	9.92b-d	8.87e	9.40B	6.11ab	5.09f	5.60 A-C	3.81b	3.78b	3.80B
HC 2%	10.37ab	9.21de	9.79AB	5.66b-e	5.35d-f	5.51BC	4.71a	3.86b	4.29 A
HC 3%	10.66ab	9.42de	10.04 A	6.02bc	5.63c-e	5.83AB	4.64a	3.79b	4.22 A
MO 2%	10.26a-c	9.13de	9.70AB	6.54a	5.23ef	5.89 A	3.72b	3.90b	3.81B
MO 3%	10.31ab	9.17de	9.74AB	5.59c-e	5.28d-f	5.44 C	4.72a	3.89b	4.31 A
HC 1.5% + MO 1.5%	10.59ab	9.37de	9.98 A	5.92bc	5.57c-e	5.75 A-C	4.67a	3.80b	4.24 A
HC 2% + MO 2%	10.74a	9.50c-e	10.12 A	6.10ab	5.70b-d	5.90 A	4.64a	3.80b	4.22 A
mean	10.41 A	9.24B		5.99 A	5.41B		4.42 A	3.83B	
2023									
control	10.07b-d	9.02e	9.55 C	6.14a-d	5.31e	5.73B	3.93de	3.71e	3.82 C
HC 2%	10.51a-c	9.49de	10.00 A-C	6.19a-c	5.74b-e	5.97AB	4.32bc	3.75e	4.04BC
HC 3%	10.91ab	9.84c-e	10.38AB	6.22ab	5.93a-d	6.08 A	4.69a	3.91de	4.30 A
MO 2%	10.40a-c	9.36de	9.88BC	6.17a-c	5.68de	5.93AB	4.23 cd	3.68e	3.96 C
MO 3%	10.47a-c	9.42de	9.95 A-C	6.18a-c	5.71c-e	5.95AB	4.29c	3.71e	4.00 C
HC 1.5% + MO 1.5%	10.86ab	9.77c-e	10.32AB	6.21ab	5.87a-d	6.04AB	4.65ab	3.90de	4.28AB
HC 2% + MO 2%	11.08a	9.90 cd	10.49 A	6.24a	6.00a-d	6.12 A	4.84a	3.90de	4.37 A
mean	10.61 A	9.54B		6.19 A	5.75B		4.42 A	3.79B	

Means in the same column followed by the same letter (s) are not significantly (*p* ≥ 0.05) different. according to Tukey’s multiple range test. Comparisons among cultivars were performed using the main‑plot error term, whereas comparisons among dormancy‑breaking agent treatments and their interactions with cultivars were performed using the sub‑plot error term

Control (water spray only), HC 2%: Spraying Hydrogen cyanamide at 2%, HC 3%: Spraying Hydrogen cyanamide at 3%, MO 2%: Spraying Mineral oil at 2%, MO 3%: Spraying Mineral oil at 3%, HC 1.5% MO 1.5%: Spraying Hydrogen cyanamide at 1.5% +Mineral oil at 1.5%, HC 2% + MO 2%: Spraying Hydrogen cyanamide at 2% +Mineral oil at 2%

**Fig. 9:**
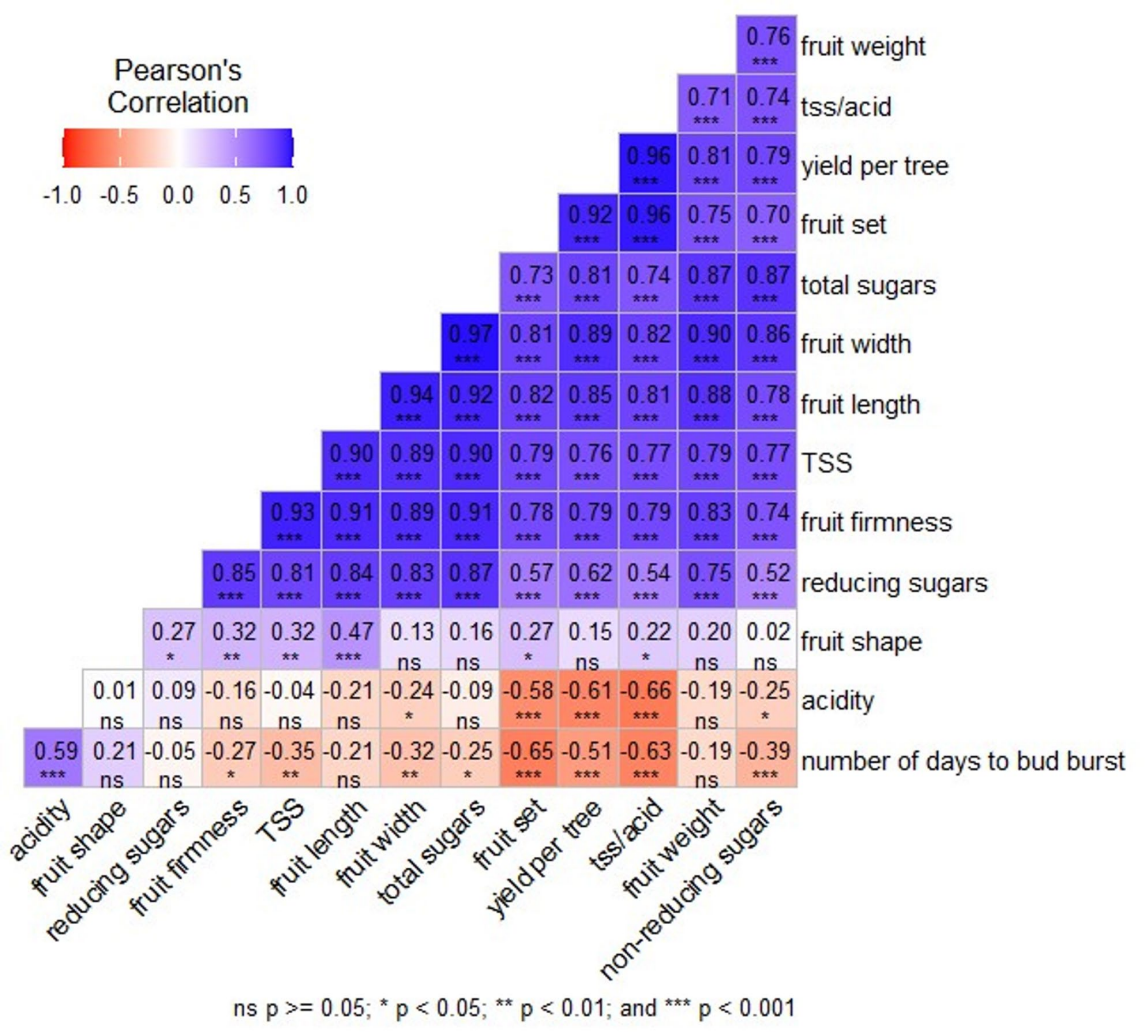
Correlations among bud break time, fruit set, fruit number per tree, yield per tree and fruit quality

Figure 9 shows the correlation matrix, which shows how bud break time (number of days to bud break) is related to fruit set, fruit number per tree, yield per tree and fruit quality. The correlation coefficients ranged from − 0.63 to 0.59, where positive values imply a direct correlation and negative values suggest an inverse relationship. Correlation analysis revealed that bud break delay was negatively affected by most productivity and fruit quality traits. Bud break exhibited a highly significant negative correlation with fruit set (*r*=-0.65, ***p < 0.001), indicating that trees exhibiting earlier bud break tended to achieve significantly greater fruit set. A similar negative trend was observed for fruit number per tree (*r*=-0.51, ***p < 0.001), although these relationships were mostly non‑significant. The yield per tree was also negatively related to bud break time, suggesting that delayed bud break was generally associated with reduced productivity, which is consistent with the strong positive correlations observed between fruit set and total yield (*r* = 0.92, **p < 0.001). In terms of fruit quality, bud break time was highly significantly negatively correlated with total sugars and non‑reducing sugars, reflecting the tendency of early‑breaking trees to accumulate relatively high sugar levels. Overall, the correlation pattern indicates that earlier bud break is generally advantageous, contributing to greater fruit set, increased fruit number and yield, and improved sugar-related fruit quality attributes. (Figure 9).

## Discussion

Deciduous fruit trees cultivated in regions with mild winters and hot, dry summers often face challenges such as inadequate bud break and a reduced proportion of blossom-bearing buds due to insufficient winter chilling. Additionally, high temperatures and low humidity during summer contribute to poor fruit set and inferior fruit quality, particularly in newly reclaimed areas of the New Valley. A practical approach to mitigate these constraints is the selection of cultivars with low chilling requirements that are well adapted to the prevailing climatic conditions [45–48]. The pear cultivars Le‑Conte and Early Gold have notably low chilling requirements, making them particularly well‑adapted to regions with mild winters. This characteristic enhances their suitability for Mediterranean or temperate zones compared with many other *Pyrus communis* cultivars, which often require higher chilling units to ensure synchronized budbreak and flowering [6, 10]. Compared with the Le‑Conte cultivar, the Early Gold cultivar presented a greater fruit set percentage and yield per tree under the conditions of this study because of its enhanced reproductive capacity and adaptability to environmental conditions, making it suitable for cultivation in the New Valley region. The successful cultivation of pear trees under relatively warm conditions is not limited to selecting the appropriate cultivar but also relies on the application of dormancy‑breaking agents that effectively stimulate the rapid and uniform release of buds from dormancy. This practice promotes synchronized bud break and enhances fruit set, ultimately improving both yield and fruit quality [35, 49]. Treatment with the dormancy-breaking agent hydrogen cyanamide (HC) helps improve bud break and increases the percentage of floral buds [47]. Moderate concentrations of hydrogen cyanamide have been reported to improve budbreak and flowering uniformity primarily in apple cultivars (e.g., ‘Eva’, ‘Gala’, and ‘Golden Delicious’) and blueberry cultivars (e.g., ‘M7’ and ‘Brigitta’), whereas excessive rates increased the risk of phytotoxicity; therefore, although similar responses have been observed in some pear cultivars (e.g., ‘Rocha’ and ‘Le‑Conte’), such effects remain cultivar‑specific and should not be directly extrapolated across species without validation [50, 51]. In the present study, combining HC with mineral oil (MO) increased bud break and floral bud percentage, leading to increased ‘Early Gold’ and ‘Le-Conte’ pear cultivar yields compared with those of individual applications, while reducing the amount of HC required [52]. In the current experiment, the superior bud break response induced by the combined HC + MO treatments was directly reflected in higher fruit set and yield values. Combining HC with mineral oil not only increased its effectiveness but also provided an economically viable alternative to using HC alone at relatively high concentrations. In addition, combination treatment improved bud metrics but also translated into increased fruit set and yields. These results reinforce the importance of selecting suitable dormancy-breaking treatments to ensure sustainable pear production in regions with inadequate winter chilling [53]. Studies have shown that, compared with HC alone, HC + MO results in superior fruit set, increasing yield, carbohydrate content, the C/N ratio, and hormone levels (IAA, GA₃). These findings indicate that earlier and more uniform bud break leads to improved reproductive performance and potentially improved fruit quality [49, 54]. Research on ‘Le-Conte’ pears in Egypt has shown that the application of dormancy-breaking agents, particularly hydrogen cyanamide (e.g., Dormex) at 1–3% mixed with 3% mineral oil, significantly improves cultivation success. When sprayed in late January or early February, this combination induces earlier and more uniform budbreak, increases flowering and fruit set percentages, and substantially increases yields compared with those of untreated trees [49, 55]. Similarly, a study on ‘Rocha’ pear reported that a mixture of 1.5% hydrogen cyanamide with 3% mineral oil increased budbreak by 40.2% compared with that of untreated trees and substantially advanced the onset of bud break [26].

In terms of fruit quality characteristics, the superior fruit quality attributes recorded in Early Gold during the present study are closely associated with its better bud break behavior and higher productivity. The superior levels of total soluble solids, sugars, and vitamin C in the Early Gold cultivar may be attributed to its enhanced photosynthetic efficiency and more effective assimilate translocation, traits commonly associated with increased carbohydrate accumulation in pear fruit quality studies. Variation in soluble solids and vitamin C among cultivars has also been shown to be strongly genotype‑dependent, further supporting these differences. Conversely, the higher acidity observed in Le‑Conte likely reflects slower organic acid degradation, a phenomenon well documented among certain pear genotypes with distinct metabolic rates that influence the acid–sugar balance [56]. With respect to the role of dormancy-breaking agents, the application of hydrogen cyanamide in combination with mineral oil has been shown to increase fruit quality in pears, primarily through its strong dormancy‑breaking effect, which improves bud break uniformity, flowering synchronization, and early fruit development [35, 57]. In Le‑Conte pear, spraying 3% hydrogen cyanamide with 3% mineral oil significantly increased flowering, fruit set, and yield, thereby creating physiological conditions that support improved fruit size and chemical quality attributes compared with those of untreated trees. Similar studies on other cultivars, such as ‘Rocha’, have demonstrated that hydrogen cyanamide combined with 3% mineral oil markedly enhances budbreak rates, advancing early development stages that indirectly contribute to higher fruit quality at harvest. Additional evidence shows that 1–2% Dormex combined with 3% mineral oil is particularly effective in increasing bud break and floral initiation in Le‑Conte, improvements that translate into better fruit development and superior quality outcomes under warm‑winter conditions. The study’s correlation patterns demonstrate how crucial bud break timing is in controlling pear tree productivity and fruit quality characteristics. Early bud break encourages more efficient flower development and subsequent fruit retention, as evidenced by the strong negative correlation found between bud break time and fruit set. This finding is in line with past findings that pear cultivars vary greatly in their phenological timing and that early initiating cultivars typically have better reproductive performance and more abundant flowering. The advantage of early bud development in maximizing fruit set was supported by [58], who showed that cultivars that started floral primordia earlier produced richer flowering and more stable reproductive behavior across years. Similarly, the negative associations between bud break time, fruit number per tree, and yield per tree in the present data align with findings that bud quality and developmental timing strongly influence yield potential [59]. reported that spur size, bud quality, and wood age significantly affect fruit set and whole‑tree yield in ‘Conference’ pear, emphasizing that earlier, well‑developed buds increase productive capacity. These physiological relationships explain why the delayed bud break observed in the present study was associated with a reduced fruit number and lower yield per tree. From a physiological perspective, the negative association between bud break timing and productive traits can be linked to mechanisms underlying dormancy release. Early bud break reflects a more efficient transition from dormancy to active growth, a process strongly influenced by hormonal and carbohydrate pathways. Recent work on pear Para dormancy indicates that auxin and carbohydrate redistribution modulate bud activation and subsequent flowering behavior [60], providing biochemical support for the association between early bud break and greater fruit set.

## Conclusion

This study demonstrated that dormancy‑breaking agents, particularly hydrogen cyanamide in combination with mineral oil, play critical roles in improving the budbreak, vegetative growth, yield, and fruit quality of pear trees grown under mild‑winter conditions. Among the treatments evaluated in this study, the combined application of 2% hydrogen cyanamide + 2% mineral oil was the most effective treatment, producing the earliest and highest bud break, the greatest fruit set, and the highest yield, while also enhancing physical and chemical fruit characteristics. Moderate concentrations of hydrogen cyanamide alone or mixed with mineral oil (3% HC and 1.5% HC + 1.5% MO) also performed well, confirming the value of integrated dormancy‑breaking strategies in warm regions.

Across treatments, ‘Early Gold’ outperformed ‘Le‑Conte’ in terms of fruit set, yield, and overall fruit quality, indicating its superior adaptability to the arid conditions of the New Valley region. Correlation analysis further highlighted the importance of early bud break as a key determinant of productivity and sugar-related fruit quality.

Overall, the findings provide strong evidence that combining hydrogen cyanamide with mineral oil offers a practical and highly effective approach to overcoming insufficient winter chilling, supporting sustainable pear production in regions affected by warming climatic trends. However, broader recommendations regarding optimal concentrations would require dose‒response experiments conducted across multiple seasons and environments.

## Data Availability

The datasets generated during the current study are available from the corresponding author on reasonable request.
